# Supplementary material for: mRNA lipid nanoparticle-mediated pyroptosis sensitizes immunologically cold tumors to checkpoint immunotherapy
Source: Nat Commun. 2023 Jul 15;14:4223. doi: 10.1038/s41467-023-39938-9 (PMC10349854; doi:10.1038/s41467-023-39938-9)
Supplement: Supplementary file 1 — Supplementary Information [file 41467_2023_39938_MOESM1_ESM.pdf]

Supplementary Information

mRNA lipid nanoparticle-mediated pyroptosis sensitizes immunologically cold tumors to checkpoint immunotherapy

Fengqiao Li[1], Xue-Qing Zhang[2,3]*, William Ho[1], Maoping Tang[2,3], Zhongyu Li[1], Lei Bu[4], and Xiaoyang Xu[1,5]*

[1]Department of Chemical and Materials Engineering, New Jersey Institute of Technology, Newark, NJ 07102, USA.

[2]Shanghai Frontiers Science Center of Drug Target Identification and Delivery, School of Pharmacy, Shanghai Jiao Tong University, 800 Dongchuan Road, Shanghai 200240, PR China.

[3]National Key Laboratory of Innovative Immunotherapy, Shanghai Jiao Tong University, Shanghai 200240, PR China.

[4]Department of Medicine, NYU Grossman School of Medicine, NY 10016, USA.

[5]Department of Biomedical Engineering, New Jersey Institute of Technology, Newark, NJ 07102, USA.

*Corresponding author. Email: xueqingzhang@sjtu.edu.cn (X.-Q. Z.); xiaoyang.xu@njit.edu (X.X.)

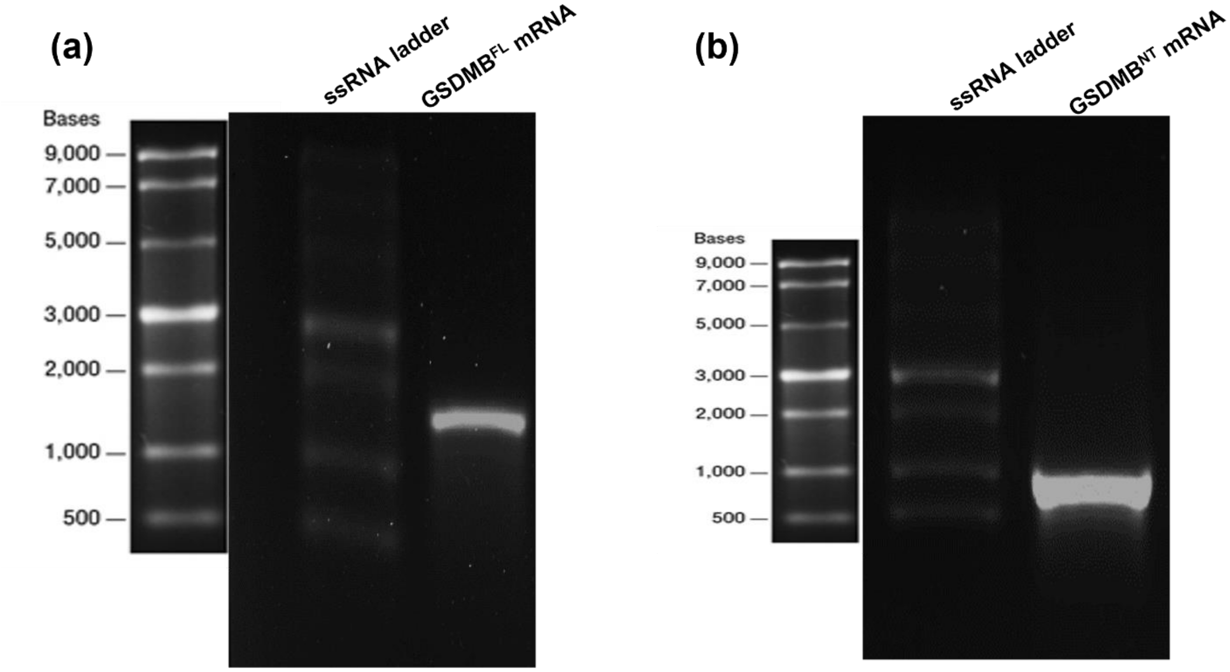

Supplementary Fig. 1 Native agarose gel electrophoresis to identify the size of synthetic mRNAs encoding GSDMB full-length (GSDMB^{FL}, **a**), and GSDMB N-terminal (GSDMB^{NT}, **b**). Data are representative of three independent experiments.

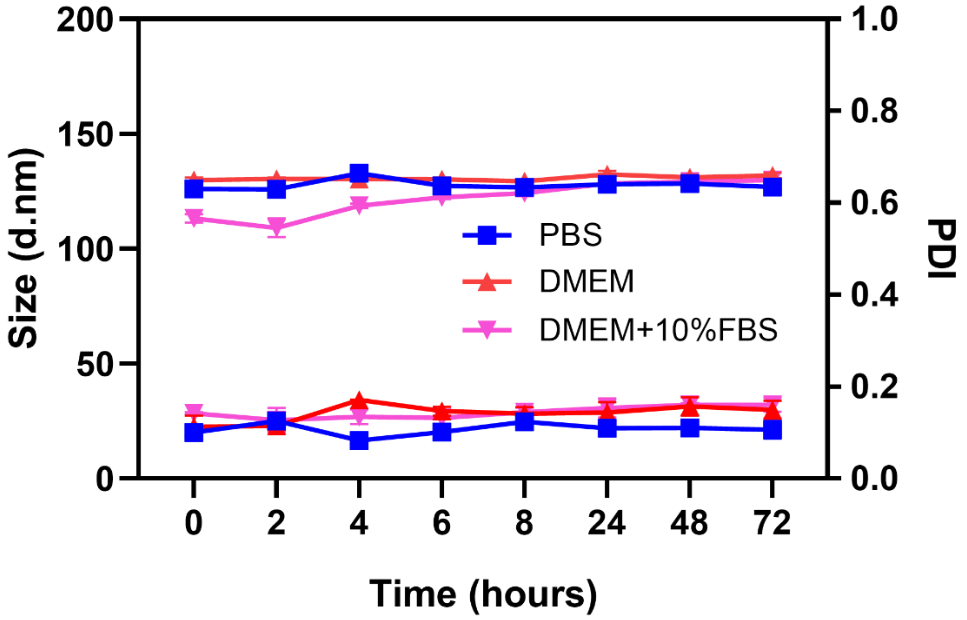

Supplementary Fig. 2 The stability of GSDMB^{NT} mRNA@LNPs in PBS, DMEM, and DMEM + 10% FBS. Data are presented as means ± SD (n = 3). Source data are provided as a Source Data file.

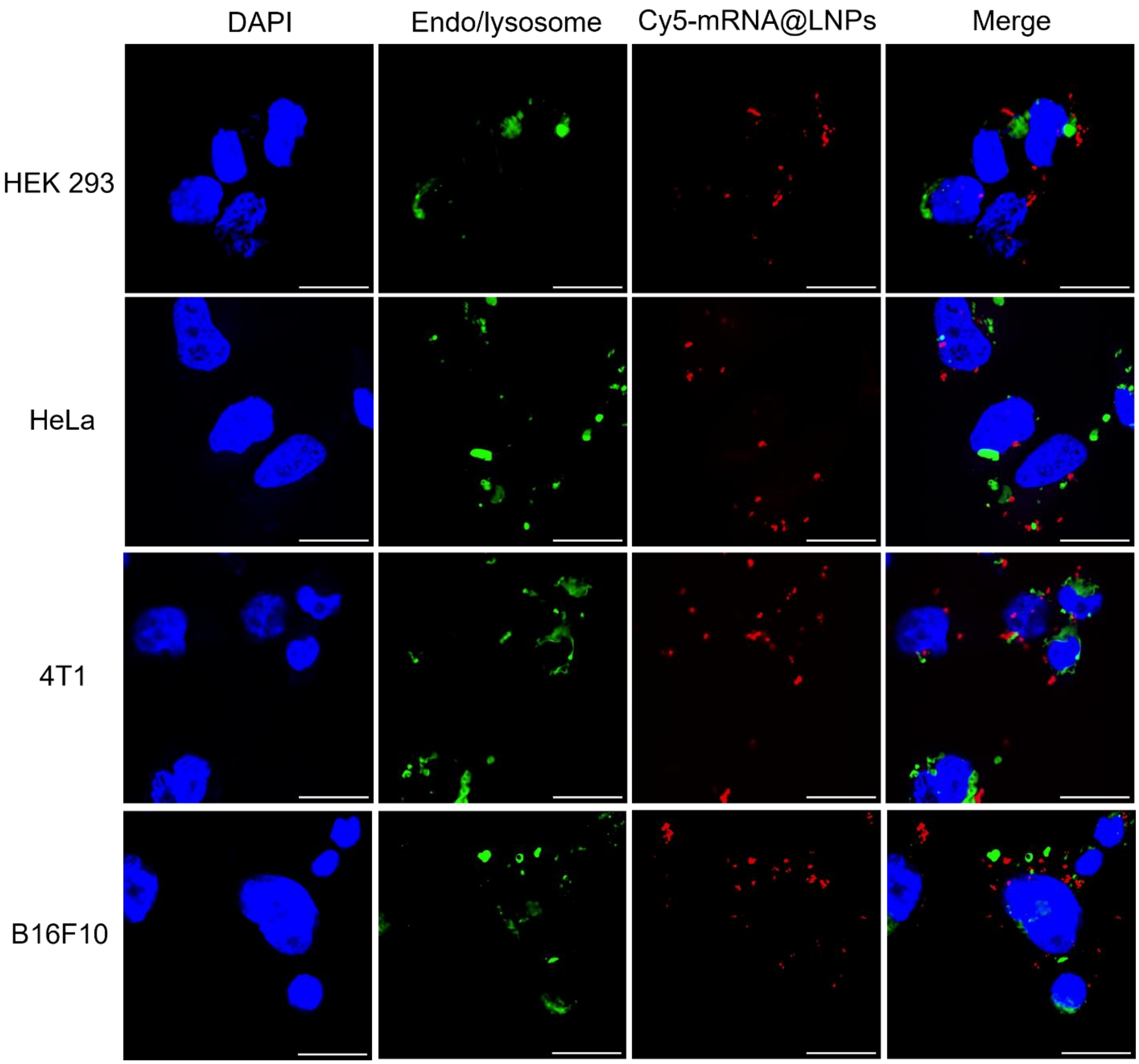

Supplementary Fig. 3 LNP-mediated endosomal/lysosomal escape and cytoplasmic release of Luc ^{Cy5}mRNA in HEK 293, HeLa, 4T1, and B16F10 cells 4 hours after incubation. DAPI (blue), Endo/lysosome (green), Luc ^{Cy5}mRNA@LNPs (red), scale bar = 20 μm. Data are representative of two independent experiments.

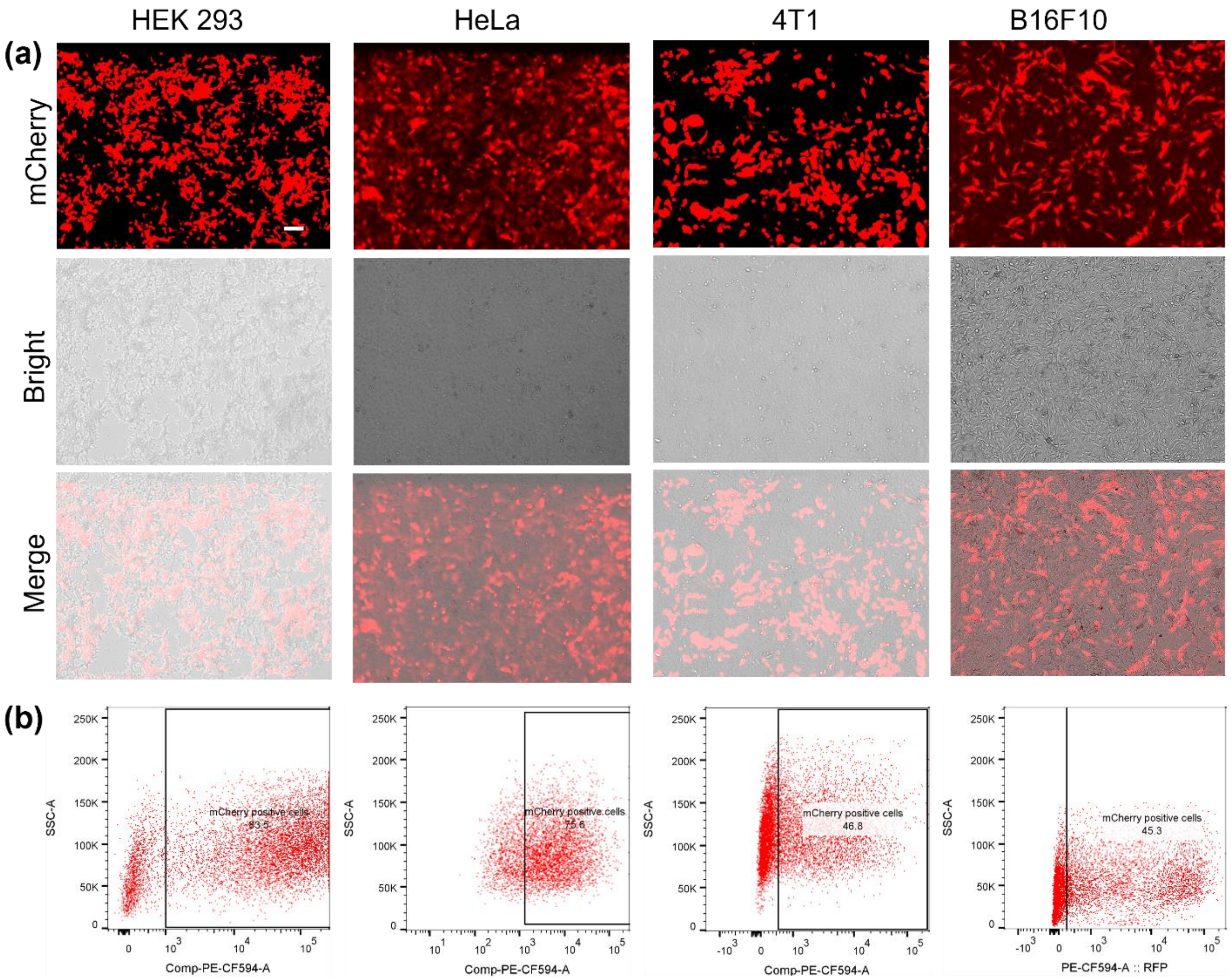

Supplementary Fig. 4 Transfection efficacy of LNPs was determined by fluorescence microscope **(a)** and flow cytometry analysis **(b,** n = 3**)** in four different cell lines. Scale bar = 100 μm. Data in **(a)** is representative of three independent experiments.

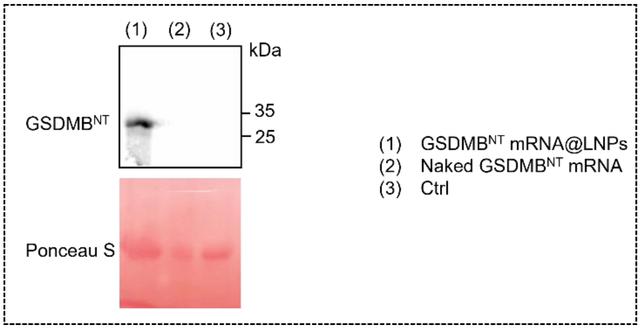

Supplementary Fig. 5 Western blot analysis of GSDMB^{NT} expression.

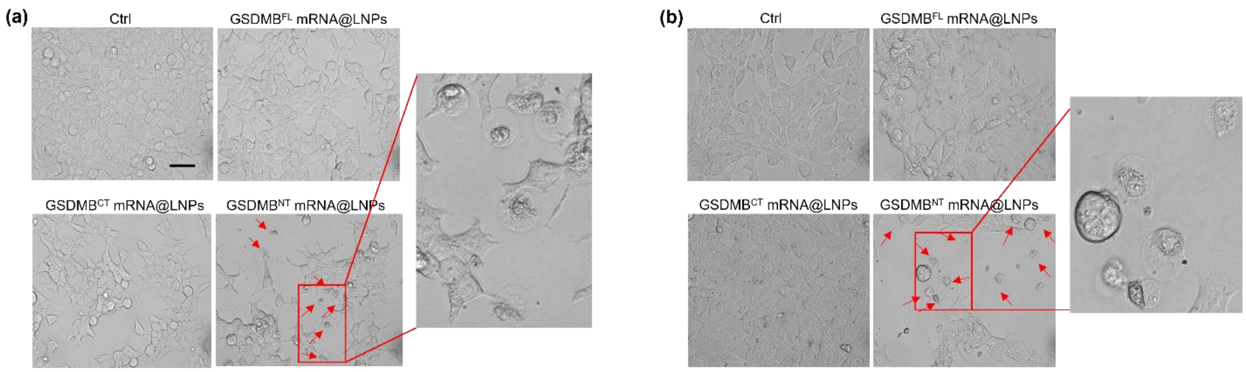

Supplementary Fig. 6 Representative images of HEK 293 **(a)** and 4T1 **(b)** cells transfected by mRNA/LNPs encoding GSDMB full-length ($GSDMB^{FL}$), GSDMB C-terminal ($GSDMB^{CT}$), or GSDMB N-terminal ($GSDMB^{NT}$) after 24 hours of treatment. Scale bar = 50 μm. Data are representative of three independent experiments.

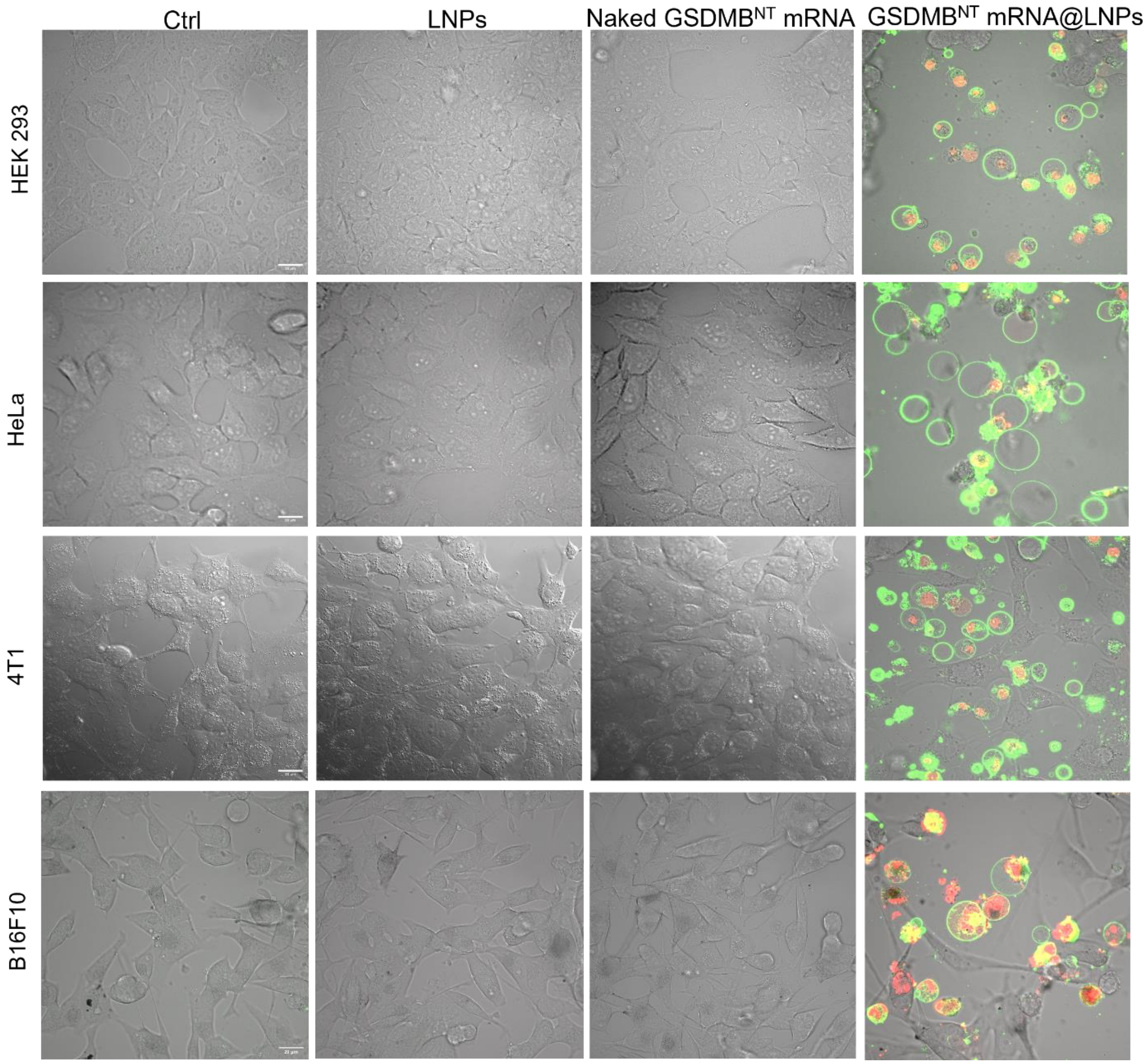

Supplementary Fig. 7 Cell morphologies of the treated HEK293, HeLa, 4T1, and B16F10 cells were detected using a confocal microscope. Before imaging, cells were added with annexin V-FITC and propidium iodide (PI) for 15 mins incubation. Scale bars = 20 μm. All data shown are representative of three independent experiments.

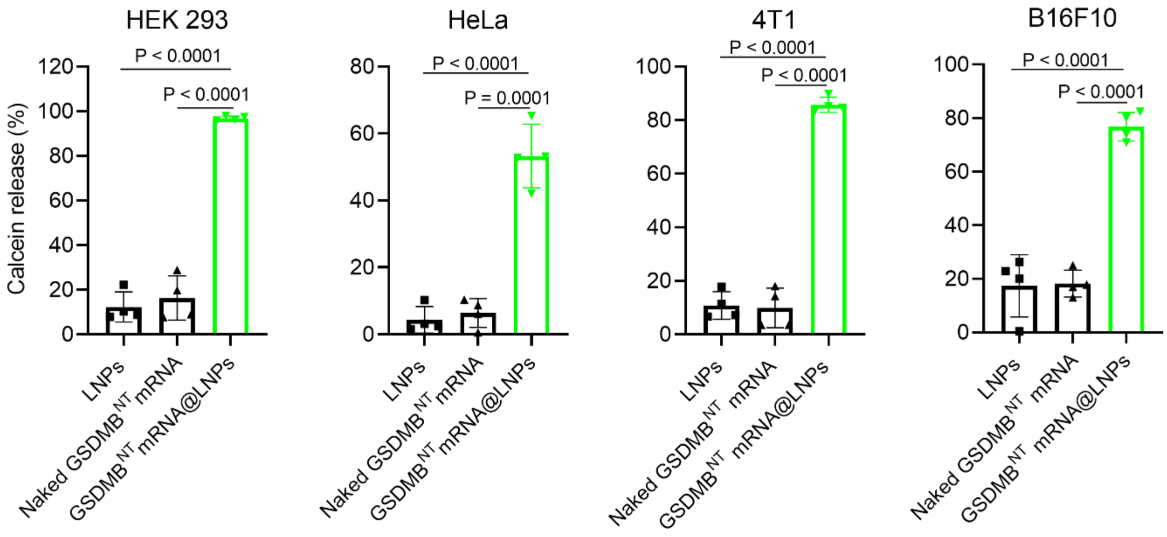

Supplementary Fig. 8 Quantification of calcein AM release. All data are presented as means ± SD (n = 4). Statistical significance was calculated using a two-tailed Student's t test. Source data are provided as a Source Data file.

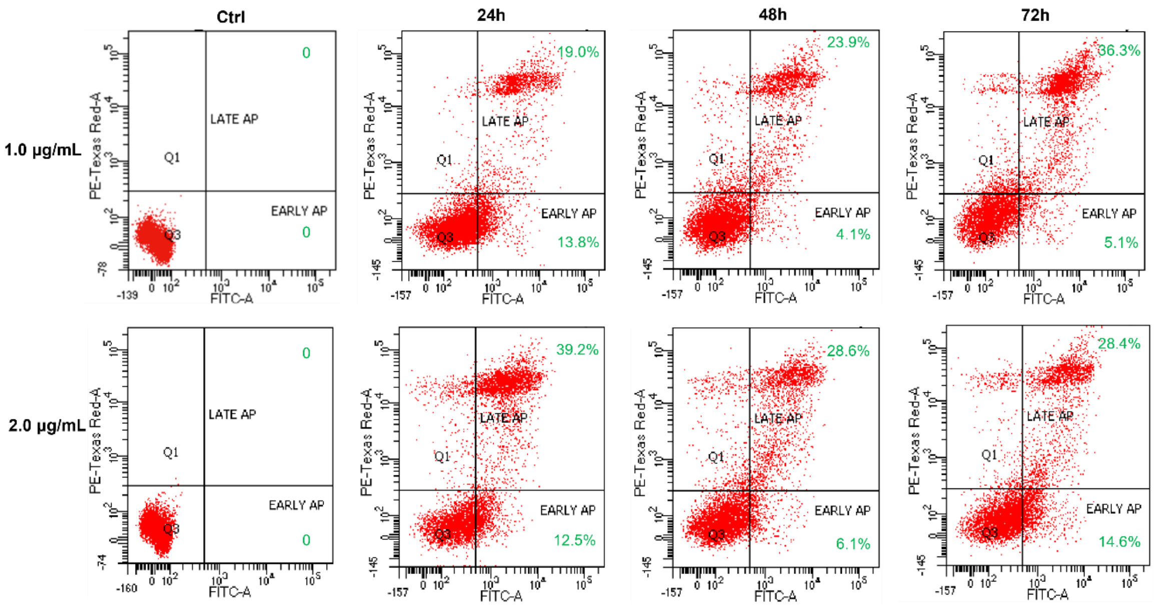

Supplementary Fig. 9 Percentage of apoptotic cells was determined by staining with FITC-Annexin V/PI (n = 3 biological replicates per group). HEK 293 cells were transfected with GSDMB^{NT} mRNA@LNPs at various mRNA concentrations for 24, 48, and 72 hours.

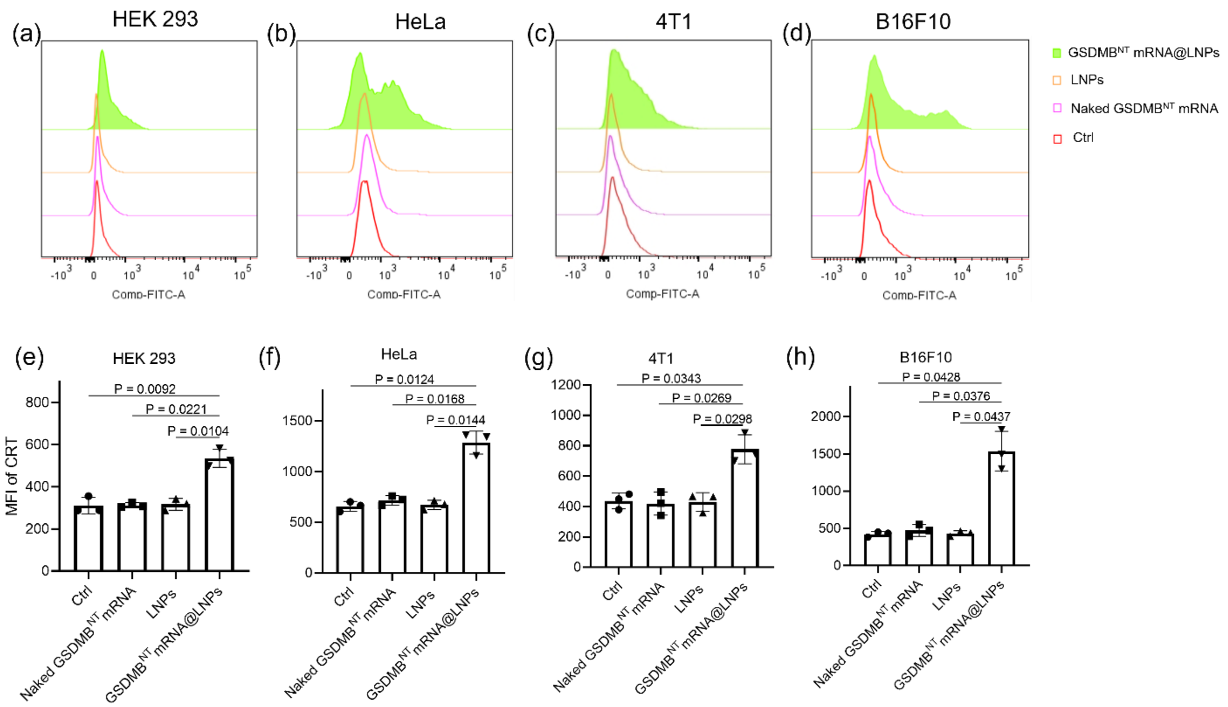

Supplementary Fig. 10 GSDMB^{NT} mRNA@LNP treatment increases calreticulin (CRT) surface exposure of HEK 293 (**a** and **e**), HeLa (**b** and **f**), 4T1 (**c** and **g**), and B16F10 (**d** and **h**) cells. Cells were treated with the indicated treatments for 48 hours, and then collected and stained with an Alexa Fluor 488-labeled CRT antibody for flow cytometry analysis. All data are presented as means ± SD (n = 3). Statistical significance was calculated via one-way ANOVA. Untreated cells served as the control (Ctrl) in all experiments. MFI: mean fluorescence intensity. Source data are provided as a Source Data file.

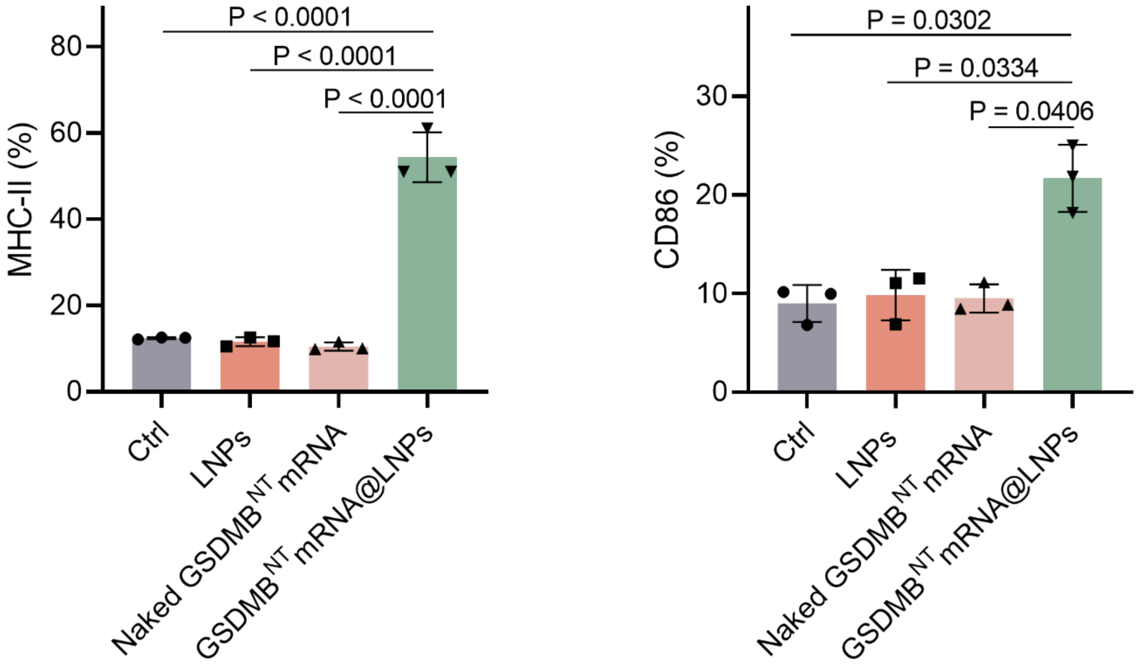

Supplementary Fig. 11 Flow cytometry analysis of DC maturation biomarkers (MHC-II and CD86). Data are presented as means ± SD (n = 3). Statistical significance was calculated via one-way ANOVA. Source data are provided as a Source Data file.

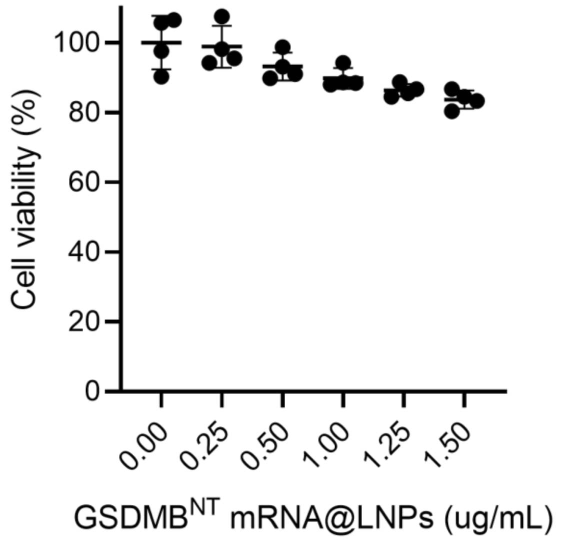

Supplementary Fig. 12 Cell viability of bone marrow-derived macrophages and DCs after treatment with GSDMB^{NT} mRNA@LNPs for 48 hours. Data are presented as means ± SD (n = 4 biological replicates per group). Source data are provided as a Source Data file.

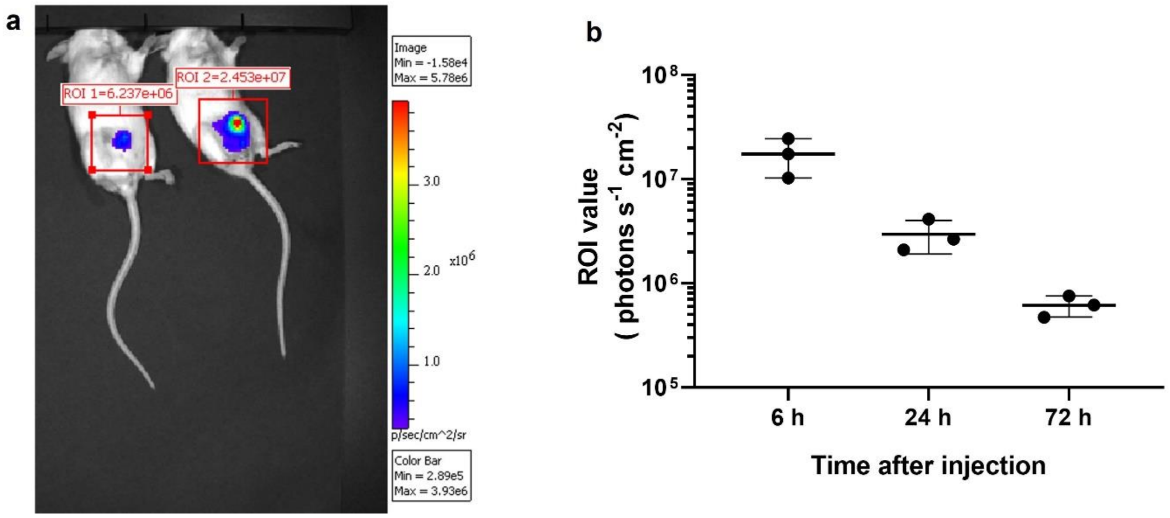

Supplementary Fig. 13 In vivo bioluminescence of luciferase mRNA-encapsulating LNPs of intratumoral injection in orthotopic 4T1 tumors. **a**, Representative images show luciferase activity after 6 hours. Anti-Reverse Cap Analog (ARCA)-capped luciferase mRNA (left) and CleanCap-capped luciferase mRNA (right). **b**, A time course of CleanCap-capped luciferase mRNA bioluminescence activity is shown as photons $s^{-1}cm^{-2}$ as values within regions of interest (ROI). Data are presented as means ± SD (n = 3 mice per group). Source data are provided as a Source Data file.

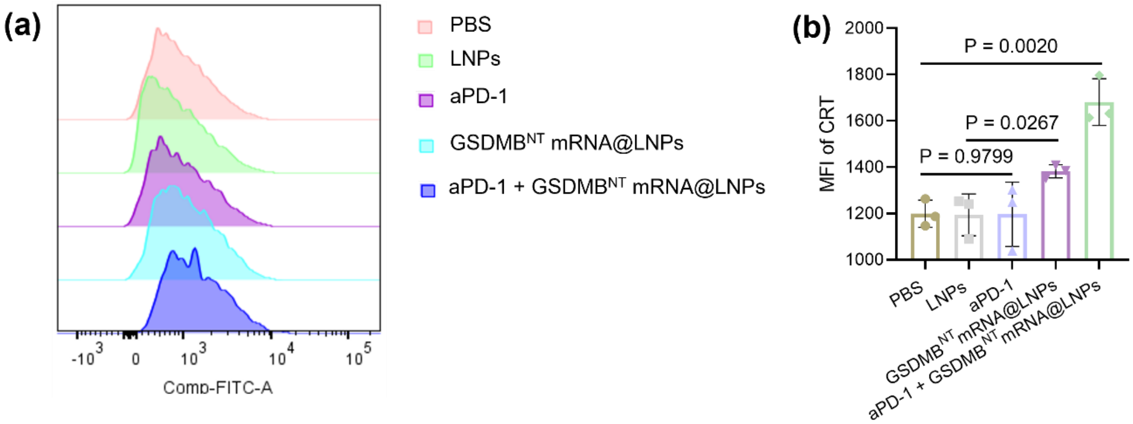

Supplementary Fig. 14 In vivo evaluation of CRT surface exposure in cells isolated from tumor tissues obtained from the 4T1-bearing mouse model. **(a)** Flow cytometry analysis of CRT-positive cells after the indicated treatments. **(b)** Histogram analysis performed by FlowJo software. Data are presented as mean ± SD (n = 3 mice per group). Statistical significance was calculated using a two-tailed Student's t test. MFI: mean fluorescence intensity. Source data are provided as a Source Data file.

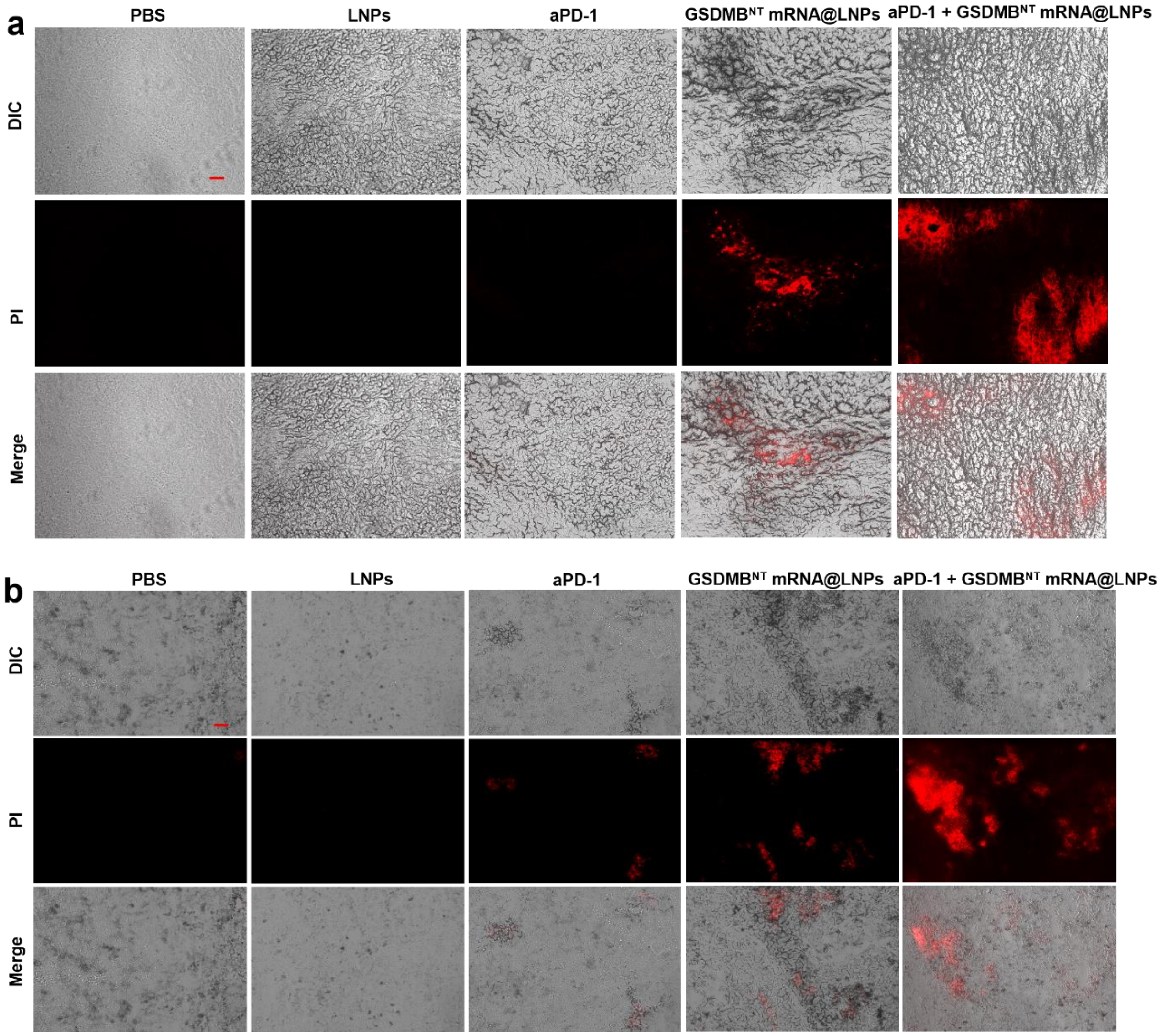

Supplementary Fig. 15 Representative tumor-section images of in vivo pyroptosis cells in the orthotopic 4T1-bearing mouse model (**a**) and B16F10-bearing mouse model (**b**), n = 3 mice per group. Propidium iodide was intravenously injected into the mice before the assay. Scale bar = 50 μm. Data are representative of three independent samples.

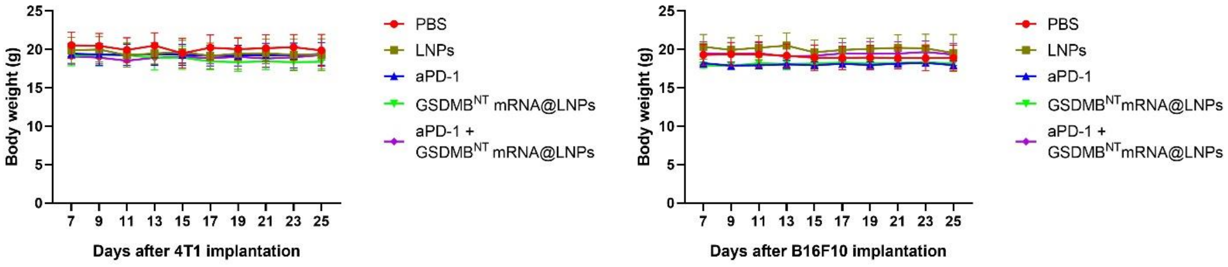

Supplementary Fig. 16 The changes in body weight in 4T1 tumor-bearing mice (left, n = 7 mice per group) and B16F10 tumor-bearing mice (right, n = 8 mice for PBS, aPD-1, or GSDMB^{NT} mRNA@LNP treatment groups, n = 7 mice for LNPs treatment group, and n = 10 mice for aPD-1 + GSDMB^{NT} mRNA@LNP treatment group) with treatments indicated. Data are presented as means ± SD. Source data are provided as a Source Data file.

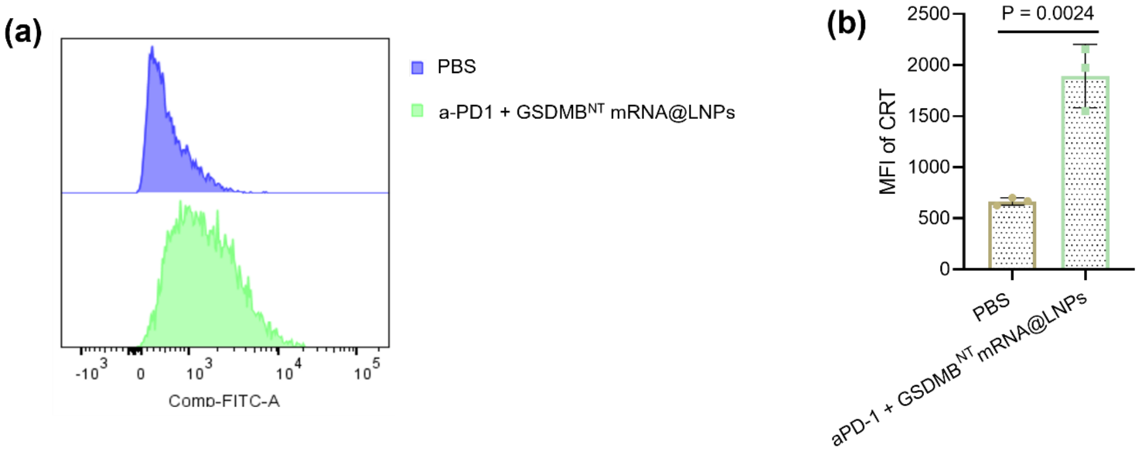

Supplementary Fig. 17 In vivo evaluation of CRT surface exposure in cells isolated from tumor tissues obtained from the B16F10-bearing mouse model. **(a)** Flow cytometry analysis of CRT-positive cells after the indicated treatments. **(b)** Histogram analysis performed by FlowJo software. Data are presented as mean ± SD (n = 3 mice per group). Statistical significance was calculated using a two-tailed Student's t test. MFI: mean fluorescence intensity. Source data are provided as a Source Data file.

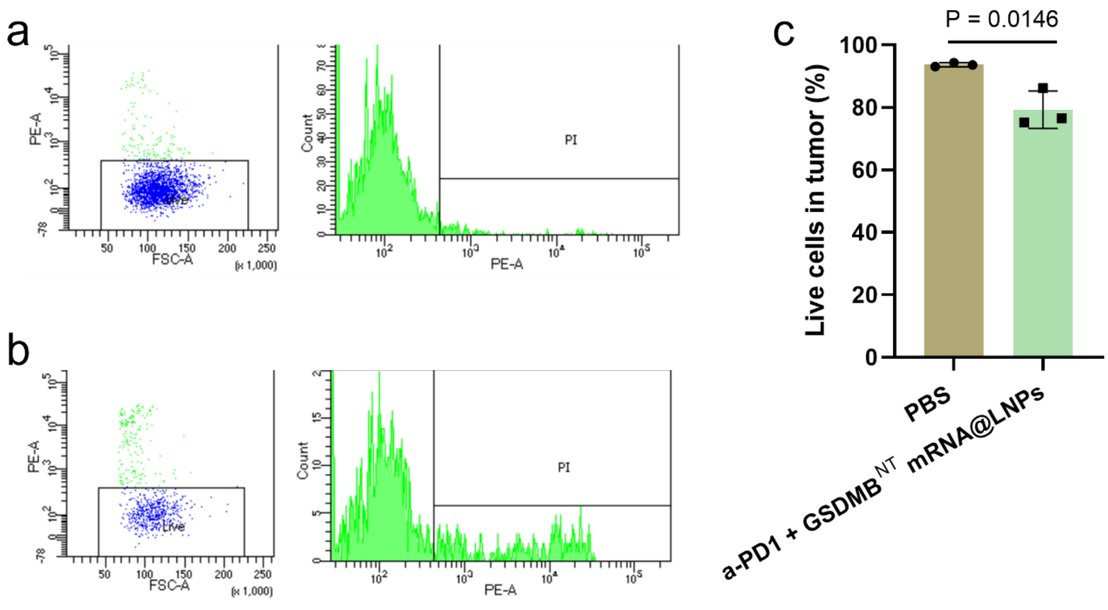

Supplementary Fig. 18 Cell viability assay in B16F10 tumors following combination treatment of aPD1 and GSDMB^{NT} mRNA@LNPs. **a**-**b**, Assessment of the population of PI-positive cells. **c**, Quantitative analysis of live cells in B16F10 tumors. Data are presented as means ± SD (n = 3 mice per group). Statistical significance was calculated using a two-tailed Student's t test. Source data are provided as a Source Data file.

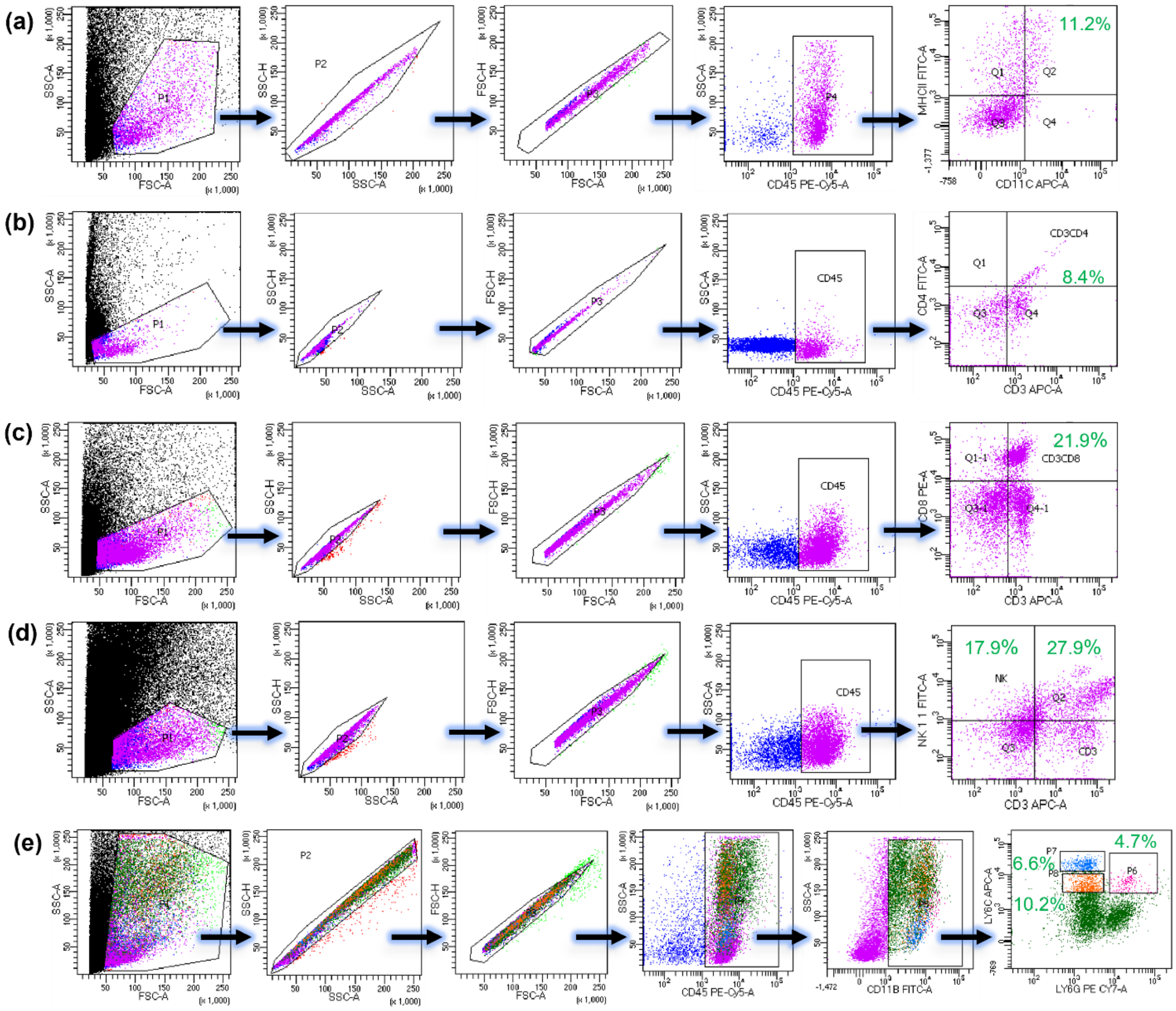

Supplementary Fig. 19 Primary gating strategy for flow cytometric analysis of dendritic cells **(a)**, CD4⁺ T cells in tumors **(b)**, CD8⁺ T cells **(c)**, NK and NK T (Q2) cells **(d)**, granulocytes (P6) **(e)**, M-MDSCs (P7), and monocyte cells (P8) in tumors. B16F10 tumor-bearing mice were euthanized, and lymph nodes were isolated on day 18. Singlet cells were selected from the cell population. CD45⁺ cells were selected from the living cell population. MHC, major histocompatibility complex; FSC-A, forward scatter area; SSC-A, side scatter area; FSC-H, forward scatter height; SSC-H, side scatter height.

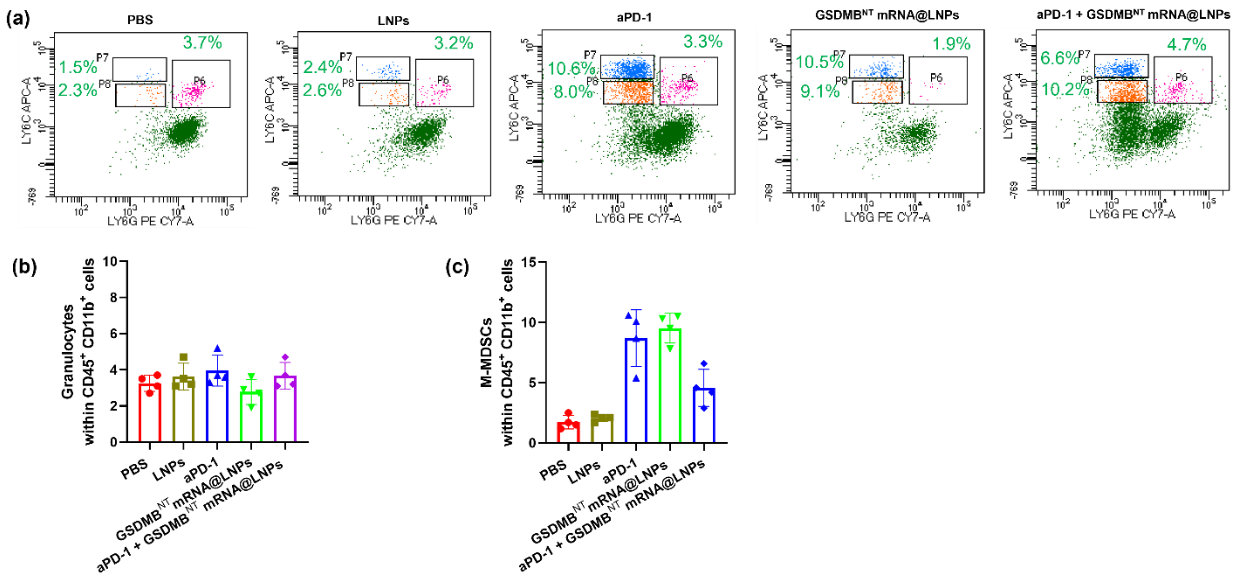

Supplementary Fig. 20 Presentative gating strategy **(a)**, quantitative analysis of granulocytes **(b)** and M-MDSCs **(c)** in tumors using flow cytometry. Data are presented as means ± SD (n = 4 mice per group). Source data are provided as a Source Data file.

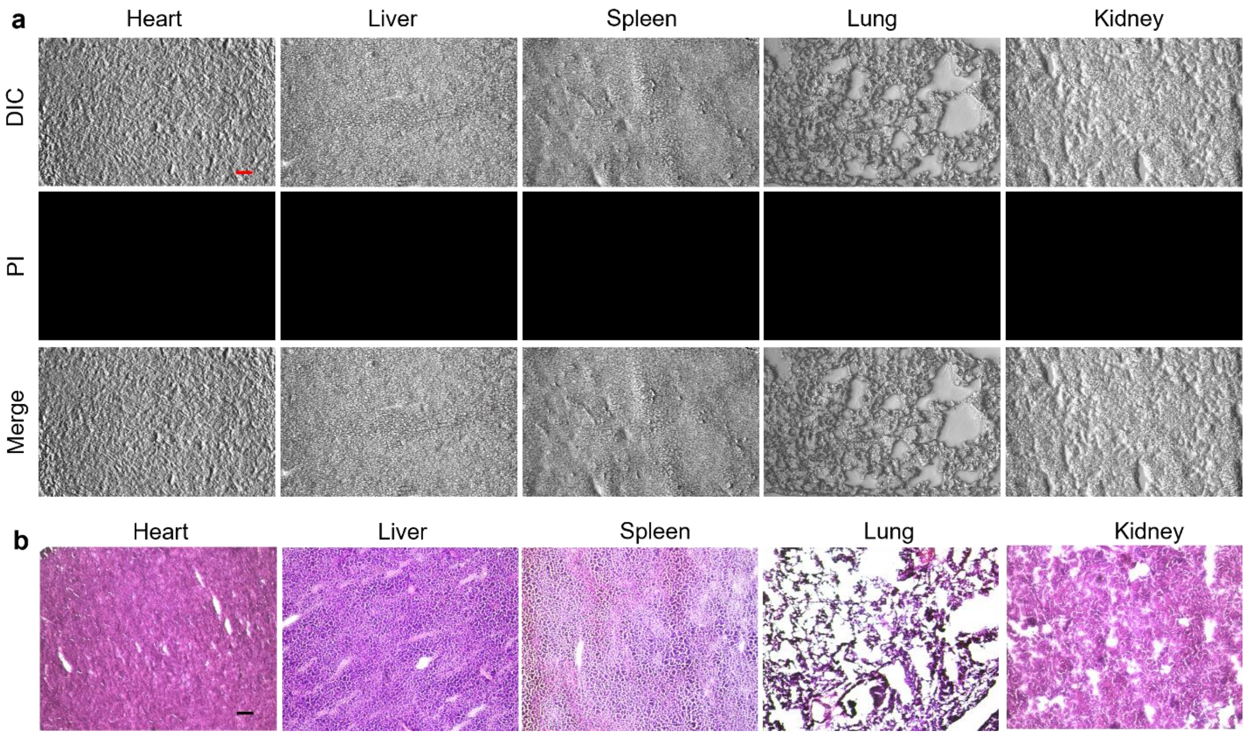

Supplementary Fig. 21 a, Representative tumor-section images of in vivo pyroptosis cells in major organs in the combinational treatment of aPD-1 and GSDMB^{NT} mRNA@LNPs in B16F10-bearing mice, n = 3 mice per group. Propidium iodide was intravenously injected into the mice before the assay. **b**, H&E staining of major organs in the combination treatment of aPD-1 and GSDMB^{NT} mRNA@LNPs in B16F10 tumor-bearing mice. Scale bar = 50 μm. Data are representative of three independent samples.

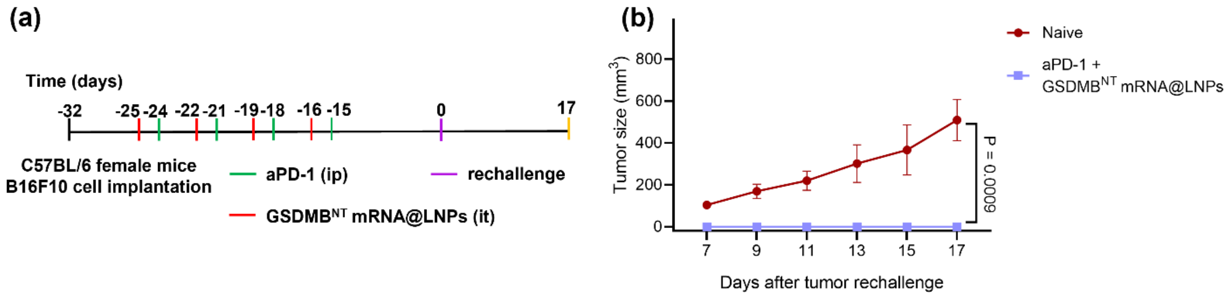

Supplementary Fig. 22 Enhanced immunological memory of $GSDMB^{NT}$ mRNA@LNPs in combination with aPD-1 using a B16F10 tumor rechallenge model. **(a)** Experimental timeline for treatment of B16F10 tumor-bearing mice and s.c. rechallenge. B16F10 tumor-bearing mice that had previously received a combination treatment regimen of aPD-1 and $GSDMB^{NT}$ mRNA@LNPs were rechallenged with 5×10^5 B16F10 cells on the left flank. Naive mice were subcutaneously implanted with the same number of B16F10 cells on day 0 to serve as a control. The volume of the rechallenged tumors was monitored every two days. **(b)** The tumor growth profile for the naive group and the combination treatment group ($GSDMB^{NT}$ mRNA@LNPs in combination with aPD-1). Data are presented as means ± SD (n = 3 mice per group). Statistical significance was calculated using a two-tailed Student's t test. Source data are provided as a Source Data file.

Supplementary Table 1: mRNA sequences used in this study.

Name	Sequences
5’ UTR	ACUAGUAUUCUUCUGGUCCCCACAGACUCAGAGAGAACCCGCC<br>ACC
3’ UTR	GCUGGAGCCUCGGUGGCCUAGCUUCUUGCCCCUUGGGCCUCC<br>CCCCAGCCCCUCCUCCCCUUCCUGCACCCGUACCCCCGUGGUC<br>UUUGAAUAAAGUCUGAGUGGGCGGC
poly(A)	AAAAAAAAAAAAAAAAAAAAAAAAAAAAAAAGCAUAUGACUAAAAA<br>AAAAAAAAAAAAAAAAAAAAAAAAAAAAAAAAAAAAAAAAAAAAAA<br>AAAAAAAAAAAAAAAAAAAAA
GSDMB^{NT} mRNA	AUGUUCAGCGUAUUUGAGGAAAUCACAAGAAUUGUAGUUAAGG<br>AGAUGGAUGCUGGAGGGGAUAUGAUUGCCGUUAGAAGCCUUG<br>UUGAUGCUGAUAGAUUCCGCUGCUUCCAUCUGGUGGGGGAGA<br>AGAGAACUUUCUUUGGAUGCCGGCACUACACAACAGGCCUCAC<br>CCUGAUGGACAUUCUGGACACAGAUGGGGACAAGUGGUUAGAU<br>GAACUGGAUUCUGGGCUCCAAGGUCAAAAGGCUGAGUUUCAAA<br>UUCUGGAUAAUGUAGACUCAACGGGAGAGUUGAUAGUGAGAUU<br>ACCCAAAGAAAUAACAAUUUCAGGCAGUUUCCAGGGCUUCCAC<br>CAUCAGAAAAUCAAGAUAUCGGAGAACCGGAUAUCCCAGCAGUA<br>UCUGGCUACCCUUGAAAACAGGAAGCUGAAGAGGGAACUACCC<br>UUUUCAUUCCGAUCAAUUAAUACGAGAGAAAACCUGUAUCUGG<br>UGACAGAAACUCUGGAGACGGUAAAGGAGGAAACCCUGAAAAG<br>CGACCGGCAAUAUAAAUUUUGGAGCCAGAUCUCUCAGGGCCAU<br>CUCAGCUAUAAACACAAGGGCCAAAGGGAAGUGACCAUCCCCC<br>CAAAUCGGGUCCUGAGCUAUCGAGUAAAGCAGCUUGUCUUCCC

	CAACAAGGAGACGAUGAAUAUUCAUUUCAGGGGCAAAACAAAAUCCUUUCCAGAAGAGAAGGAUGGUGCUUCAUCCUGUUUAGGAAAGUAA
GSDMB^{CT} mRNA	AUGUCUUUGGGUUCGGAGGAUUCCAGAAACAUGAAGGAGAAGUUGGAGGACAUGGAGAGUGUCCUCAAGGACCUGACAGAGGAGAAGAGAAAAGAUGUGCUAAACUCCCUCGCUAAGUGCCUCGGCAAGGAGGAUAUUCGGCAGGAUCUAGAGCAAAGAGUAUCUGAGGUCCUGAUUUCCGGGGAGCUACACAUGGAGGACCCAGACAAGCCUCUCCUAAGCAGCCUUUUUAAUGCUGCUGGGGUCUUGGUAGAAGCGCGUGCAAAAGCCAUUCUGGACUUCCUGGAUGCCCUGCUAGAGCUGUCUGAAGAGCAGCAGUUUGUGGCUGAGGCCCUGGAGAAGGGGACCCUUCCUCUGUUGAAGGACCAGGUGAAAUCUGUCAUGGAGCAGAACUGGGAUGAGCUGGCCAGCAGUCCUCCUGACAUGGACUAUGACCCUGAGGCACGAAUUCUCUGUGCGCUGUAUGUUGUUGUCUCUAUCCUGCUGGAGCUGGCUGAGGGGCCUACCUCUGUCUCUUCCGAUUACAAGGAUGACGACGAUAAGUAA
GSDMB^{FL} mRNA	AUGUUCAGCGUAUUUGAGGAAAUCACAAGAAUUGUAGUUAAGGAGAUGGAUGCUGGAGGGGAUAUGAUUGCCGUUAGAAGCCUUGUUGAUGCUGAUAGAUUCCGCUGCUUCCAUCUGGUGGGGGAGAAGAGAACUUUCUUUGGAUGCCGGCACUACACAACAGGCCUCACCCUGAUGGACAUUCUGGACACAGAUGGGGACAAGUGGUUAGAUGAACUGGAUUCUGGGCUCCAAGGUCAAAAGGCUGAGUUUCAAAUUCUGGAUAAUGUAGACUCAACGGGAGAGUUGAUAGUGAGAUUACCCAAAGAAAUAACAAUUUCAGGCAGUUUCCAGGGCUUCCACCAUCAGAAAAUCAAGAUAUCGGAGAACCGGAUAUCCCAGCAGUA

	UCUGGCUACCCUUGAAAACAGGAAGCUGAAGAGGGAACUACCC<br>UUUUCAUUCCGAUCAAUUAAUACGAGAGAAAACCUGUAUCUGG<br>UGACAGAAACUCUGGAGACGGUAAAGGAGGAAACCCUGAAAAG<br>CGACCGGCAAUAUAAAUUUUGGAGCCAGAUCUCUCAGGGCCAU<br>CUCAGCUAUAAACACAAGGGCCAAAGGGAAGUGACCAUCCCCC<br>CAAAUCGGGUCCUGAGCUAUCGAGUAAAGCAGCUUGUCUUCCC<br>CAACAAGGAGACGAUGAAUAUUCAUUUCAGGGGCAAAACAAAAU<br>CCUUUCCAGAAGAGAAGGAUGGUGCUUCAUCCUGUUUAGGAAA<br>GUCUUUGGGUUCGGAGGAUUCCAGAAACAUGAAGGAGAAGUUG<br>GAGGACAUGGAGAGUGUCCUCAAGGACCUGACAGAGGAGAAGA<br>GAAAAGAUGUGCUAAACUCCCUCGCUAAGUGCCUCGGCAAGGA<br>GGAUAUUCGGCAGGAUCUAGAGCAAAGAGUAUCUGAGGUCCUG<br>AUUUCCGGGGAGCUACACAUGGAGGACCCAGACAAGCCUCUCC<br>UAAGCAGCCUUUUUAAUGCUGCUGGGGUCUUGGUAGAAGCGC<br>GUGCAAAAGCCAUUCUGGACUUCCUGGAUGCCCUGCUAGAGCU<br>GUCUGAAGAGCAGCAGUUUGUGGCUGAGGCCCUGGAGAAGGG<br>GACCCUUCCUCUGUUGAAGGACCAGGUGAAAUCUGUCAUGGAG<br>CAGAACUGGGAUGAGCUGGCCAGCAGUCCUCCUGACAUGGACU<br>AUGACCCUGAGGCACGAAUUCUCUGUGCGCUGUAUGUUGUUGU<br>CUCUAUCCUGCUGGAGCUGGCUGAGGGGCCUACCUCUGUCUC<br>UUCCGAUUACAAGGAUGACGACGAUAAGUAA

Supplementary Table 2: Antibodies used in this study.

Antibody	Clone	Conjugation	Company (Cat#)	Dilution
anti-mouse CD16/32, mAb	93	unconjugated	BioLegend (101302)	1:100
anti-mouse CD45.2, mAb	104	PerCP/Cyanine5.5	BioLegend (109828)	1:100
anti-mouse CD11c, mAb	N418	APC	BioLegend (117310)	1:100
anti-mouse I-A/I-E, mAb	M5/114.15.2	FITC	BioLegend (107605)	1:100
anti-mouse CD3, mAb	17A2	APC	BioLegend (100236)	1:100
anti-mouse CD4, mAb	GK1.5	FITC	BioLegend (100405)	1:100
anti-mouse CD8a, mAb	53-6.7	PE	BioLegend (100708)	1:100
anti-mouse NK-1.1, mAb	PK136	FITC	BioLegend (108706)	1:100
anti-mouse/human CD11b, mAb	M1/70	FITC	BioLegend (101206)	1:100
anti-mouse Ly-6C, mAb	HK1.4	APC	BioLegend (128015)	1:100
anti-mouse Ly-6G, mAb	1A8	PE/Cyanine7	BioLegend (127617)	1:100
anti-mouse CD86, mAb	GL-1	APC	BioLegend (105011)	1:100
rabbit monoclonal [EPR3924] to Calreticulin- ER Marker	EPR3924	Alexa Fluor® 488	Abcam (ab196158)	1:50
rabbit polyclonal to Calreticulin- ER Marker		unconjugated	Abcam (ab2907)	1:500
rabbit monoclonal [EPR21769] to CD8 alpha	EPR21769	unconjugated	Abcam (ab217344)	1:500
rabbit monoclonal [EPR20841] to GSDMB	EPR20841	unconjugated	Abcam (ab215729)	1:1000

mAb, monoclonal antibody.
