# Supplementary material for: mRNA lipid nanoparticle-mediated pyroptosis sensitizes immunologically cold tumors to checkpoint immunotherapy
Source: Nat Commun. 2023 Jul 15;14:4223. doi: 10.1038/s41467-023-39938-9 (PMC10349854; doi:10.1038/s41467-023-39938-9)
Supplement: Supplementary file 3 — Reporting Summary [file 41467_2023_39938_MOESM3_ESM.pdf]

nature portfolio

Corresponding author(s): Xiaoyang Xu; Xue-Qing Zhang

Last updated by author(s): Jun 11, 2023

Reporting Summary

Nature Portfolio wishes to improve the reproducibility of the work that we publish. This form provides structure for consistency and transparency in reporting. For further information on Nature Portfolio policies, see our Editorial Policies and the Editorial Policy Checklist.

Statistics

For all statistical analyses, confirm that the following items are present in the figure legend, table legend, main text, or Methods section.

n/a	Confirmed	
☐	☒	The exact sample size (n) for each experimental group/condition, given as a discrete number and unit of measurement
☐	☒	A statement on whether measurements were taken from distinct samples or whether the same sample was measured repeatedly
☐	☒	The statistical test(s) used AND whether they are one- or two-sided<br>*Only common tests should be described solely by name; describe more complex techniques in the Methods section.*
☒	☐	A description of all covariates tested
☒	☐	A description of any assumptions or corrections, such as tests of normality and adjustment for multiple comparisons
☐	☒	A full description of the statistical parameters including central tendency (e.g. means) or other basic estimates (e.g. regression coefficient) AND variation (e.g. standard deviation) or associated estimates of uncertainty (e.g. confidence intervals)
☐	☒	For null hypothesis testing, the test statistic (e.g. F, t, r) with confidence intervals, effect sizes, degrees of freedom and P value noted<br>*Give P values as exact values whenever suitable.*
☒	☐	For Bayesian analysis, information on the choice of priors and Markov chain Monte Carlo settings
☒	☐	For hierarchical and complex designs, identification of the appropriate level for tests and full reporting of outcomes
☒	☐	Estimates of effect sizes (e.g. Cohen's d, Pearson's r), indicating how they were calculated

Our web collection on statistics for biologists contains articles on many of the points above.

Software and code

Policy information about availability of computer code

Data collection	The CDS sequences of GSDMB N terminal domain, C terminal domain and full length were collected from the database of National Center for Biotechnology Information (NCBI). The sequence information in this study was included in Supplementary Table 1.
Data analysis	All results are analyzed using GraphPad Prism software and presented as the means ± SD. Unpaired t-test and one-way ANOVA were used for two-group or multiple-group comparisons. The details of statistical analysis for figures and Supplementary Figures are performed as indicated in the figure legends, and survival analysis was analyzed using the log-rank test.

For manuscripts utilizing custom algorithms or software that are central to the research but not yet described in published literature, software must be made available to editors and reviewers. We strongly encourage code deposition in a community repository (e.g. GitHub). See the Nature Portfolio guidelines for submitting code & software for further information.

Data

Policy information about availability of data

All manuscripts must include a data availability statement. This statement should provide the following information, where applicable:

- Accession codes, unique identifiers, or web links for publicly available datasets
- A description of any restrictions on data availability
- For clinical datasets or third party data, please ensure that the statement adheres to our policy

All data generated or analyzed during this study are included in this published article and its Supplementary Information file and the Source Data file. Source data are provided with this paper.

Human research participants

Policy information about studies involving human research participants and Sex and Gender in Research.

Reporting on sex and gender	N/A
Population characteristics	N/A
Recruitment	N/A
Ethics oversight	N/A

Note that full information on the approval of the study protocol must also be provided in the manuscript.

Field-specific reporting

Please select the one below that is the best fit for your research. If you are not sure, read the appropriate sections before making your selection.

- [x] Life sciences
- [ ] Behavioural & social sciences
- [ ] Ecological, evolutionary & environmental sciences

For a reference copy of the document with all sections, see nature.com/documents/nr-reporting-summary-flat.pdf

Life sciences study design

All studies must disclose on these points even when the disclosure is negative.

Sample size	The sample size (n>=3) of each experiment is included in the corresponding figure legends. At least 7 mice per group were used in the survival experiment. Sample sizes were selected to ensure that they are sufficient for statistical comparison between different experimental groups.
Data exclusions	No data were excluded from analysis.
Replication	All experiments were performed as technical or biological replications (n >= 3) as appropriate for the experiment design. Details of experimental replicates are given in the figure legends.
Randomization	No formal randomization method was used.
Blinding	Investigators were not blinded to group allocation.

Reporting for specific materials, systems and methods

We require information from authors about some types of materials, experimental systems and methods used in many studies. Here, indicate whether each material, system or method listed is relevant to your study. If you are not sure if a list item applies to your research, read the appropriate section before selecting a response.

Materials & experimental systems

n/a	Involved in the study	
	x	Antibodies
	x	Eukaryotic cell lines
x		Palaeontology and archaeology
	x	Animals and other organisms
x		Clinical data
x		Dual use research of concern

Methods

n/a	Involved in the study	
x		ChIP-seq
	x	Flow cytometry
x		MRI-based neuroimaging

Antibodies

Antibodies used	Purified anti-mouse CD16/32 antibody (clone 93), PerCP/Cyanine5.5 anti-mouse CD45.2 Antibody (clone 104), APC anti-mouse CD11c Antibody (clone N418), FITC anti-mouse I-A/I-E Antibody (clone M5/114.15.2), APC anti-mouse CD3 Antibody (clone 17A2), FITC anti-mouse CD4 Antibody (clone GK1.5), PE anti-mouse CD8a Antibody (clone 53-6.7), FITC anti-mouse NK-1.1 Antibody (clone PK136), FITC anti-mouse/human CD11b Antibody (clone M1/70), APC anti-mouse Ly-6C Antibody (clone HK1.4),PE/Cyanine7 anti-mouse Ly-6G Antibody (clone 1A8), and APC anti-mouse CD86 Antibody (clone GL-1) were purchased from BioLegend. GSDMB antibody (ab215729), Rabbit polyclonal to Calreticulin- ER Marker antiobody (ab2907), Rabbit monoclonal [EPR21769] to CD8 alpha (ab217344), and Alexa Fluor® 488 Calreticulin antibody (ab196158) were purchased from Abcam.
Validation	The validation of antibodies used in this study was included in Supplementary Table 2.

Eukaryotic cell lines

Policy information about cell lines and Sex and Gender in Research

Cell line source(s)	HEK 293, HeLa, 4T1, and B16F10-Luc cell lines were obtained from the American Type Culture Collection (ATCC). BMDCs and macrophages obtained from female C57BL/6 mice.
Authentication	None of the cell lines used were authenticated
Mycoplasma contamination	Cell lines were not tested for mycoplasma contamination
Commonly misidentified lines (See ICLAC register)	No commonly misidentified cell lines were used in the study

Animals and other research organisms

Policy information about studies involving animals; ARRIVE guidelines recommended for reporting animal research, and Sex and Gender in Research

Laboratory animals	Balb/c female mice and C57BL/6 female mice aged 6-8 weeks were purchased from the Jackson Laboratory
Wild animals	No wild animals were used.
Reporting on sex	The experiment was designed without considering the sex of the mice, and female mice were selected to ensure gender uniformity.
Field-collected samples	No field-collected samples were used.
Ethics oversight	Animal studies were reviewed and approved by the Institutional Animal Care and Use Committee of Rutgers University.

Note that full information on the approval of the study protocol must also be provided in the manuscript.

Flow Cytometry

Plots

Confirm that:

- [x] The axis labels state the marker and fluorochrome used (e.g. CD4-FITC).
- [x] The axis scales are clearly visible. Include numbers along axes only for bottom left plot of group (a 'group' is an analysis of identical markers).
- [ ] All plots are contour plots with outliers or pseudocolor plots.
- [x] A numerical value for number of cells or percentage (with statistics) is provided.

Methodology

Sample preparation	Tumors were harvested and digested by 1 mg/mL collagenase IV (Thermo Fisher Scientific) for 30 mins at 37 °C to make single-cell suspensions. The single-cell suspensions were then passed through 70-µm nylon cell strainers. The suspension was centrifuged, and the cell pellets were washed and resuspended in the PBS containing 1% BSA (FACS buffer) and stained with the indicated antibodies for another 1 hour.
Instrument	Samples were analyzed using a FACS analyzer (BD Biosciences, San Jose, CA).
Software	All flow cytometry data were analyzed using FlowJo software.
Cell population abundance	No cell sorting was performed.
Gating strategy	The preliminary FSC/SSC gates were determined by the blank cell samples.

☒ Tick this box to confirm that a figure exemplifying the gating strategy is provided in the Supplementary Information.
